# Visual and Anatomical Outcomes of Branch Retinal Vein Occlusion in Routine Care: A Retrospective Study of 102 Eye Records

**DOI:** 10.7759/cureus.119647

**Published:** 2026-11-14

**Authors:** Suki Bernstein, Cora Berdejo

**Affiliations:** 1 Emergency Medicine, George Eliot Hospital NHS Trust, Nuneaton, GBR; 2 Ophthalmology, Warwickshire Hospital, Warwick, GBR

**Keywords:** anti-vegf treatment, branch retinal vein occlusion, macular oedema, oct (optical coherence tomography), retrospective research, visual acuity measurement

## Abstract

Background

Branch retinal vein occlusion (BRVO) can cause visual loss through macular oedema. Routine-care outcomes reflect heterogeneous treatment histories and follow-up. This study evaluated visual and anatomical changes and their association in a district-hospital BRVO cohort.

Methods

We retrospectively analysed 102 eye records classified into 11 treatment pathways within a five-year period from January 2019 to December 2023. Visual acuity (VA), recorded in letters, and five-field average optical coherence tomography (OCT) thickness were compared between baseline and the last recorded appointment with a minimum of four weeks of follow-up. Mean changes were calculated with 95% confidence intervals (CIs). Pearson and Spearman correlations assessed the association between VA gain and OCT thickness reduction. Treatment groups were described without causal comparisons.

Results

Median follow-up was 23 months (interquartile range, 12-38.5; range, 2-73). Mean VA increased from 60.0 ± 17.0 to 72.3 ± 13.8 letters; mean change was 12.3 ± 16.7 letters (95% CI, 9.0-15.5). Mean five-field average OCT thickness declined from 414.5 ± 109.5 to 300.5 ± 55.7 µm; mean reduction was 113.9 ± 125.2 µm (95% CI, 89.4-138.5). Forty-two eye records (41.2%) gained at least 15 letters, and four (3.9%) lost at least 15 letters. VA gain correlated with thickness reduction (Pearson r = 0.54; 95% CI, 0.38-0.66; Spearman ρ = 0.59). Exclusion of two zero-letter baseline records yielded a mean gain of 11.8 ± 16.5 letters. The aflibercept and ranibizumab categories comprised 35 and 30 records, respectively.

Conclusions

Visual improvement and reduced five-field average OCT thickness were observed during routine BRVO care, with a moderate association between functional and anatomical change. Ranibizumab showed the largest mean VA gain amongst the single-agent treatment groups, exceeding the observed aflibercept. Variable follow-up, small treatment subgroups, and non-random treatment selection limit comparative interpretation. The findings support assessing vision and retinal anatomy together and do not establish superiority of any treatment pathway.

## Introduction

Branch retinal vein occlusion (BRVO) is an important cause of visual impairment associated with retinal vascular disease. Venous obstruction can produce retinal haemorrhage, macular oedema, and impaired perfusion. Management aims to preserve visual function, control oedema, and identify complications, including retinal neovascularisation [1].

Randomised trials have established the efficacy of intravitreal anti-vascular endothelial growth factor (anti-VEGF) therapy for BRVO-associated macular oedema. BRAVO demonstrated greater visual gains with ranibizumab than sham treatment, whilst VIBRANT demonstrated better visual and anatomical outcomes with aflibercept than macular grid laser [2,3]. BRIGHTER evaluated individualised ranibizumab treatment with or without laser and found no additional functional benefit or reduction in injection requirements from adding laser [4]. Dexamethasone intravitreal implant provides another treatment option, with selection influenced by ocular risk factors and anticipated treatment burden [1,5].

Outcomes from protocol-driven trials do not fully describe the experience of routine services. In clinical practice, patients enter treatment with different visual potential, receive variable numbers of injections, and may change therapy over time. Long-term observational studies and extension studies demonstrate that some eyes require ongoing treatment several years after presentation [6,7]. Describing local outcomes is therefore useful, provided that the endpoint and treatment history are defined and differences between pathways are not interpreted as randomised comparisons.

This study examined visual and anatomical outcomes in a district-hospital BRVO cohort using individual eye records. The principal objective was to estimate the change in visual acuity (VA) between baseline and the last recorded appointment in eyes with BRVO-associated macular oedema. Secondary objectives were to quantify change in five-field average optical coherence tomography (OCT) thickness, examine its association with VA change, and describe the distribution of outcomes across recorded treatment pathways.

## Materials and methods

Study design, setting, and governance

This was a retrospective observational analysis of BRVO care at a district general hospital in Warwickshire, UK. The original project was described as covering 2019-2023; however, recorded follow-up extended to 73 months. We acknowledge the wide range of follow-up durations. However, the median follow-up was 23 months, with the middle 50% of patients followed for 12-38.5 months, indicating that the cohort included substantial longitudinal observation. Comparing baseline and final measurements within each eye allowed us to assess the observed functional and anatomical changes during that eye’s documented clinical course. Nevertheless, this paired approach does not eliminate the influence of differing follow-up durations. The findings should therefore be interpreted as outcomes at the last available clinical assessment, rather than estimates of treatment efficacy at a uniform time point. We have made this distinction explicit and acknowledged variable follow-up as a limitation. Outcomes were analysed at each record’s last available appointment, rather than at a common five-year endpoint. Reporting was structured with reference to the Strengthening the Reporting of Observational Studies in Epidemiology (STROBE) recommendations [8].

Consent of participants was gained, and ethics approval was sought but not needed for this study. The source provided for analysis contained no names, dates of birth, or hospital identifiers. No assumption of ethics exemption was made from the retrospective design alone.

Participants and data source

The analytical dataset comprised all 102 eye records in the project’s tabulated data file. The original project materials specified BRVO and the exclusion of glaucoma, diabetes, and other ocular diseases such as age-related macular degeneration.

BRVO-associated macular oedema at baseline was a mandatory inclusion criterion. Macular oedema was identified from documented baseline OCT findings, interpreted in the context of the clinical diagnosis of BRVO. OCT thickness was used to quantify anatomical change rather than as the sole diagnostic criterion.

Cataract was not an exclusion criterion because it does not directly cause the macular oedema associated with BRVO, and its inclusion allowed the cohort to reflect routine clinical practice. By contrast, age-related macular degeneration, diabetic retinopathy, and glaucoma were excluded as alternative retinal or optic nerve conditions that could confound the assessment of visual outcomes.

Cases were identified electronically from the electronic records of clinic lists and departmental register over a five-year period. Eligibility required documented diagnosis of BRVO by the treating ophthalmologist. Diagnostic ascertainment relied on the recorded clinical diagnosis rather than independent retrospective adjudication of retinal images. VA and five-field average OCT thickness were extracted at baseline and the last recorded follow-up appointment. The sampling was consecutive, done through manual screening of records. VA was extracted from clinical records.

No records were removed from the supplied dataset, and the VA and OCT endpoints and recorded follow-up were complete for all 102 records. Completeness of this extract does not establish completeness of the underlying clinical cohort.

Each row was treated as one eye record. Patient linkage identifiers were unavailable; consequently, uniqueness across treatment categories and inclusion of both eyes from the same patient could not be independently verified. Interval estimates assume independent observations and require recalculation with patient-level clustering if bilateral records are present.

Variables and treatment pathways

Recorded variables were laterality, superior or inferior BRVO location, gender as labelled in the source, current age, treatment category, injection count, baseline and last-appointment VA, baseline and last-appointment five-field average OCT thickness, and follow-up in months. The source age field was labelled ‘current age’; it was therefore not treated as age at diagnosis. Baseline was labelled as the day of first injection or diagnosis. Baseline was defined as the assessment at the first intravitreal injection visit, from which the initial VA and OCT measurements were obtained. This represents the start of treatment and should not be interpreted as the onset of symptoms.

The date of symptom onset was not consistently documented in the retrospective clinical records; therefore, the interval between symptom onset and treatment initiation could not be reliably determined. We acknowledge this as a limitation, since differences in disease duration before treatment may have influenced baseline findings and subsequent outcomes.

VA was analysed on the recorded letter scale without further conversion. The OCT variable was retained as five-field average thickness in micrometres; it was not relabelled as central subfield thickness. VA gain was calculated as final minus baseline VA, and anatomical reduction as baseline minus final OCT thickness. Positive values therefore represented improvement in either measure, although reduced thickness alone does not establish resolution of oedema or recovery of retinal function.

We acknowledge that central subfield thickness (CST) is the conventional anatomical outcome used in BRVO studies. In this retrospective study, five-field average macular thickness was analysed because it was the measurement consistently available in the clinical records at baseline and follow-up.

The Royal College of Ophthalmologists guideline supports OCT for assessing macular oedema and treatment response, although it does not specifically recommend five-field averaging. Our choice of measurement therefore reflected the available clinical data. Using the same measurement at both time points enabled assessment of within-eye changes in this anatomical parameter.

We recognise that five-field average thickness is not interchangeable with CST. Averaging across several fields may attenuate changes confined to the central subfield and limit direct comparison with studies reporting CST. Our anatomical findings should therefore be interpreted as changes in five-field average macular thickness, alongside the VA outcomes.

The 11 source treatment categories were retained. Brand names were standardised to bevacizumab, aflibercept, ranibizumab, and dexamethasone implant. The category labelled ‘combi anti-VEGF’ was described as multiple anti-VEGF exposure; the specific agents, sequence, and timing were not available. A plus sign denotes treatments recorded within a pathway and does not establish concurrent administration. Laser type and indication were unavailable. Injection entries of the form ‘16 + 4’ were summed for a descriptive total, whilst laser sessions were excluded from injection counts. The completeness of implant counts in other mixed pathways could not be established; these totals were not used to estimate annualised treatment intensity.

Statistical analysis

Analyses were developed for this retrospective reanalysis of the existing dataset; no prospectively registered statistical analysis plan was available. Continuous variables were summarised using means and standard deviations (SDs), or medians and interquartile ranges (IQRs). Categorical variables were reported as counts and percentages. All available records were included without imputation. The sample size was determined by the available cohort, without a formal a priori power calculation.

Mean paired changes were estimated using Student t-based 95% confidence intervals (CIs). Wilson CIs were calculated for the proportions gaining or losing at least 15 letters. These thresholds describe the recorded letter scale and depend on confirmation of its measurement method. Pearson correlation, with a Fisher-transformed 95% CI, assessed the association between VA gain and OCT thickness reduction. Spearman correlation was used to assess robustness to non-normal distributions and extreme observations. Correlations were exploratory and do not imply that anatomical change caused visual change.

Two post hoc sensitivity analyses examined the influence of excluding records with baseline VA recorded as zero letters and restricting analysis to follow-up of at least 12 months. The latter remained an analysis of variable last-visit outcomes, not a 12-month endpoint. Treatment groups were summarised descriptively; no treatment-effect hypothesis tests or multivariable superiority models were fitted because most groups were very small and treatment selection and sequencing were incompletely characterised. Calculations used Python with NumPy 2.3.5 and SciPy 1.17.0 (Python Software Foundation, Fredericksburg, VA, USA). All 204 recalculated change values agreed with the source’s derived columns; totals for the three largest treatment categories were also reconciled.

## Results

Cohort characteristics

The extract included 102 eye records across 11 recorded treatment pathways. Source-recorded current age was 73.3 ± 10.9 years (range, 41-96). There were 54 male-labelled records (52.9%) and 48 female-labelled records (47.1%). Left eyes accounted for 53 records (52.0%), and superior BRVO for 58 (56.9%). Median follow-up was 23 months (IQR, 12-38.5; range, 2-73); 80 records (78.4%) had at least 12 months of follow-up. All 102 rows had baseline and last-visit VA and OCT values (Table 1).

**Table 1:** Characteristics and overall outcomes of the 102 eye records Current age is the source-recorded age, not confirmed baseline age. Injection counts sum explicit components and exclude laser sessions; completeness of mixed-pathway counts requires verification. BRVO: branch retinal vein occlusion; VA: visual acuity; OCT: optical coherence tomography; SD: standard deviation; IQR: interquartile range; CI: confidence interval

Variable	Value
Current age (years), mean (SD)	73.3 (10.9)
Male/female, n (%)	54 (52.9)/48 (47.1)
Left/right eye, n (%)	53 (52.0)/49 (48.0)
Superior/inferior BRVO, n (%)	58 (56.9)/44 (43.1)
Follow-up (months), median (IQR)	23 (12-38.5)
Recorded injections, median (IQR)	7 (4-12)
Baseline/last-visit VA, letters, mean (SD)	60.0 (17.0)/72.3 (13.8)
VA gain, letters, mean (95% CI)	12.3 (9.0-15.5)
Baseline/last-visit five-field OCT thickness (µm), mean (SD)	414.5 (109.5)/300.5 (55.7)
OCT thickness reduction (µm), mean (95% CI)	113.9 (89.4-138.5)

Visual and anatomical outcomes

Mean VA increased from 60.0 ± 17.0 to 72.3 ± 13.8 letters, giving a paired mean gain of 12.3 ± 16.7 letters (95% CI, 9.0-15.5). Median gain was 11.5 letters (IQR, 4-21); individual changes ranged from a loss of 46 to a gain of 51 letters. Forty-two records gained at least 15 letters (41.2%; 95% CI, 32.1%-50.9%), and four lost at least 15 letters (3.9%; 95% CI, 1.5%-9.7%). Any gain was recorded for 80 eyes (78.4%).

Mean five-field average OCT thickness decreased from 414.5 ± 109.5 to 300.5 ± 55.7 µm. Mean paired thickness reduction was 113.9 ± 125.2 µm (95% CI, 89.4-138.5); median reduction was 73 µm (IQR, 37-169). Individual changes ranged from an increase of 143 µm to a reduction of 654 µm. Thickness decreased in 94 records (92.2%). VA gain and reduction in five-field average thickness were moderately correlated (Pearson r = 0.54; 95% CI, 0.38-0.66; Spearman ρ = 0.59; Figure 1).

**Figure 1:**
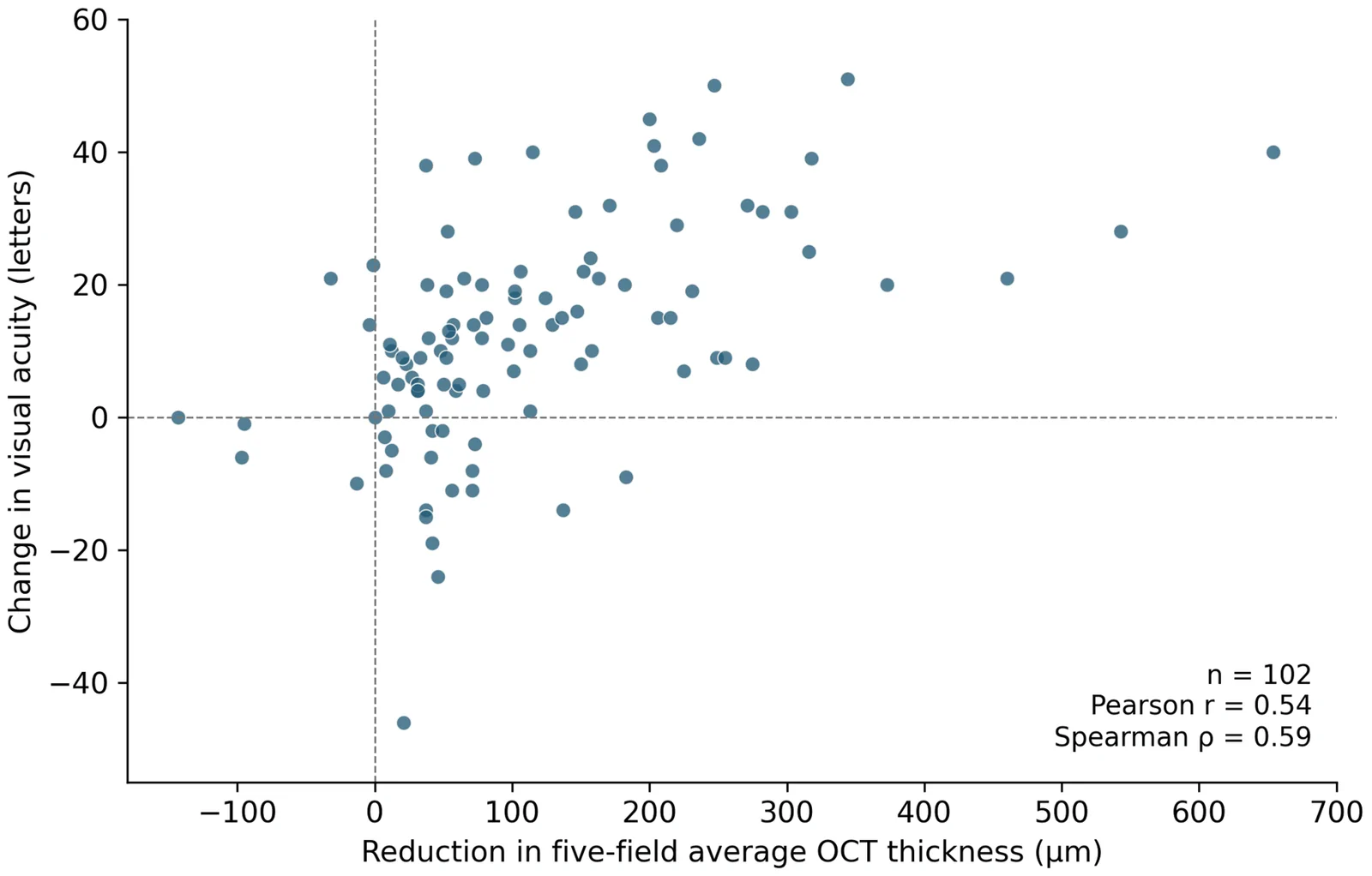
Association between anatomical and visual change at the last recorded appointment Each point represents one of 102 eye records. Thickness reduction is baseline minus last-visit five-field average OCT thickness; VA change is last-visit minus baseline letter score. Dashed lines indicate no change. Follow-up ranged from 2 to 73 months. The association is descriptive and does not represent a causal treatment effect. OCT: optical coherence tomography; VA: visual acuity

Recorded treatment pathways

Aflibercept (n = 35), ranibizumab (n = 30), and multiple anti-VEGF exposure (n = 15) accounted for 80 of 102 records (78.4%). Other categories contained one to seven records each. Unadjusted VA gains were 10.6 ± 16.5, 16.8 ± 15.8, and 15.6 ± 18.3 letters in those three groups, respectively (Table 2). Baseline VA was 62.1 ± 13.6 and 56.5 ± 18.7 letters in the aflibercept and ranibizumab groups, respectively. Treatment choice, baseline vision, duration, and switching were not exchangeable across pathways; these descriptive values cannot rank drug efficacy.

**Table 2:** Descriptive outcomes by recorded treatment pathway A plus sign indicates recorded exposure within a pathway, without confirmed timing or sequence. Positive changes indicate VA gain or thickness reduction; negative changes indicate VA loss or thickness increase. Categories are descriptive and are not estimates of comparative treatment efficacy. ¹Letters; baseline VA is the mean. ²Mean (SD); SD is not shown for single records. ³Five-field average OCT thickness (µm). ⁴Median (IQR) (months); single values for n = 1. DEX: dexamethasone intravitreal implant; VEGF: vascular endothelial growth factor; VA: visual acuity; OCT: optical coherence tomography; SD: standard deviation; IQR: interquartile range

Pathway	n	Baseline VA¹	VA gain¹^,^²	OCT reduction²^,^³	Follow-up⁴
Bevacizumab	2	77.0	2.5 (23.3)	134.0 (137.2)	25 (19-31)
Aflibercept	35	62.1	10.6 (16.5)	115.7 (123.8)	19 (12-38.5)
Aflibercept + DEX	3	63.0	4.7 (16.5)	13.0 (163.5)	47 (33-53.5)
Ranibizumab	30	56.5	16.8 (15.8)	117.9 (140.0)	17 (6.25-28.5)
Dexamethasone	2	75.0	-6.5 (4.9)	-3.0 (14.1)	17.5 (17.25-17.75)
Multiple anti-VEGF	15	59.6	15.6 (18.3)	150.1 (125.8)	29 (24-37)
Multiple anti-VEGF + DEX	7	55.1	1.3 (17.2)	87.3 (87.3)	33 (31-45.5)
Laser	3	75.7	5.0 (5.6)	14.0 (15.7)	21 (13-34.5)
Laser + anti-VEGF	3	58.7	14.0 (6.6)	159.3 (105.2)	33 (18.5-53)
Laser + anti-VEGF + DEX	1	37.0	38.0	208.0	49
Laser + multiple anti-VEGF + DEX	1	42.0	31.0	146.0	70

The median recorded injection count was seven (IQR, 4-12; range, 0-32). Aflibercept and ranibizumab records had mean counts of 7.4 and 6.2, respectively, across their entire recorded follow-up. Steroid-containing and laser-containing pathways were too small and heterogeneous for reliable comparative conclusions. No systematic adverse-event fields were available, so safety rates could not be estimated.

Sensitivity analyses

Two records had baseline VA entered as zero letters. Removing both left 100 records, with mean VA gain of 11.8 ± 16.5 letters (95% CI, 8.5-15.1) and mean thickness reduction of 106.9 ± 112.7 µm (95% CI, 84.5-129.2). Correlation remained positive (Pearson r = 0.52; 95% CI, 0.36-0.65; Spearman ρ = 0.56). In 80 records with at least 12 months of follow-up, mean VA gain was 12.0 ± 17.0 letters (95% CI, 8.2-15.8) and mean thickness reduction was 110.0 ± 107.8 µm (95% CI, 86.0-134.0). This is not a fixed 12-month outcome analysis.

## Discussion

Principal findings

The principal observation was a 12.3 ± 16.7-letter gain in VA, accompanied by a 113.9 ± 125.2 µm reduction in five-field average OCT thickness, across 102 BRVO eye records at varying last-visit dates. At least 15 letters of improvement occurred in 42 records (41.2%), but four records (3.9%) lost at least 15 letters. The distribution was wide, indicating that favourable group-level averages conceal very different courses in individual eyes. The mean effects were similar in sensitivity analyses excluding two zero-letter baselines and restricting the sample to longer recorded follow-up, although neither sensitivity analysis resolves selection bias or missing patient linkage.

An important contribution of the study is that its endpoints were recalculated from the 102 individual rows rather than estimated by averaging treatment-group summaries. Because the groups contain between one and 35 rows, averaging category means equally would over-represent the smallest categories. The cohort-wide paired results, their SDs, and their CIs therefore describe the observed sample more faithfully. However, they do not isolate the effects of treatment from the natural disease course, regression to the mean, changes in measurement method, or differential follow-up.

Several limitations constrain interpretation of these findings. The retrospective, single-centre design introduces potential selection and information bias and limits generalisability to other clinical settings. Without a suitable control group, the observed improvements cannot be attributed solely to treatment, as natural history, regression to the mean, and concurrent ocular changes may also contribute. Treatment-specific outcomes are particularly susceptible to confounding by indication: baseline VA, disease severity, duration of macular oedema, previous response, and treatment exposure may have influenced both pathway selection and subsequent outcomes. Small, heterogeneous subgroups and incomplete documentation of administration schedules and treatment sequencing further limit meaningful comparisons.

Consequently, the greater observed visual gain in the ranibizumab subgroup represents an unadjusted descriptive finding and should not be interpreted as evidence of superiority. Follow-up ranging from two to 73 months also means that outcomes reflect different stages of disease and treatment; attendance, discharge, and loss to follow-up may influence the final recorded measurements. Furthermore, the absence of patient-level linkage prevented verification of bilateral inclusion, and any unaccounted inter-eye correlation could lead to underestimated uncertainty. Although CIs and sensitivity analyses strengthen the presentation, they do not resolve these potential systematic biases. The findings therefore describe visual and anatomical changes within this cohort and their association, without establishing causal treatment effects or comparative effectiveness.

Relationship to existing evidence

These findings align in direction with the established efficacy of anti-VEGF therapy in BRVO, including the BRAVO ranibizumab trial and the VIBRANT aflibercept trial [2,3]. Those studies had controlled eligibility criteria, treatment schedules, and specified assessment windows, unlike this service-based extract. Direct comparisons of magnitudes are therefore inappropriate. In a large UK multicentre electronic-record cohort, Gale and colleagues found sustained improvements in recorded VA with anti-VEGF therapy in routine BRVO care, whilst highlighting substantial attrition during longer observation [9]. Ciulla and colleagues similarly analysed 15,613 retinal vein occlusion eyes in a US database and identified a relationship between treatment intensity and visual outcomes, with baseline VA ceiling effects [10]. These reports reinforce the importance of the presentation VA, visit structure, and total treatment exposure missing from our adjusted comparison.

A direct real-world registry comparison of 322 BRVO eyes by Hunt and colleagues reported similar adjusted 12-month VA outcomes for ranibizumab and aflibercept, despite differing anatomical responses and switching frequencies [11]. That context is particularly relevant here: unadjusted gains were 16.8 ± 15.8 letters in the 30 ranibizumab records versus 10.6 ± 16.5 in the 35 aflibercept records, but the ranibizumab records started with lower VA and were observed for different durations. We therefore cannot infer ranibizumab superiority or use switching categories as though they were randomised treatment arms. The last recorded visit in this dataset occurred between two and 73 months; it is not equivalent to the uniform 12-month endpoint used in the registry.

Functional and anatomical change

Mean five-field average OCT thickness fell by 113.9 ± 125.2 µm, and its reduction correlated moderately with VA improvement (Pearson r = 0.54; Spearman ρ = 0.59). This provides internally consistent functional and anatomical signals, but the association is not deterministic: comparable anatomical reductions accompanied different VA changes in Figure 1. The study did not include macular ischaemia status, ellipsoid-zone integrity, other retinal structural markers, or cataract progression, each of which could help explain discordance. Crucially, correlations of two observed changes cannot establish that reduction in retinal thickness caused visual recovery.

Our OCT endpoint is explicitly a five-field average, not central subfield thickness. CST is widely used in comparative BRVO trials and registries; pooling or directly contrasting its numerical changes with a five-field average would be misleading. Nor can a declining average thickness, on its own, establish absence of persistent intraretinal fluid, preserved retinal structure, or durable remission. Clear documentation of OCT device, scanning field, and segmentation quality is needed to make these data reproducible.

Treatment pathways and clinical implications

Treatment pathways were descriptive labels accumulated over follow-up, not baseline assignments. The 30 ranibizumab records showed a 16.8 ± 15.8-letter gain, compared with 10.6 ± 16.5 letters in the 35 aflibercept records. Because the groups had differing baseline VA (56.5 ± 18.7 and 62.1 ± 13.6 letters, respectively) and non-standardised follow-up, these results cannot establish a drug-specific effect. Switching and addition of dexamethasone are also potentially markers of persistent or more complex disease, rather than independent exposures. Group sizes, clinical indication, and missing time-stamped treatment sequences would prevent reliable causal adjustment even if a formal comparative model was fitted.

Laser-containing categories were too small and heterogeneous to estimate additional benefits. The randomised BRIGHTER study found no clear visual or treatment-burden advantage from adding laser to an individualised ranibizumab regimen [4]. In a separate real-world retrospective comparison, Thomley and colleagues likewise found no additional VA or injection-number advantage from adding focal laser to anti-VEGF treatment [12]. These external findings do not resolve the different indications for laser in our cohort, including treatment of non-perfusion or neovascularisation. Similarly, two dexamethasone-only eye records with worse VA cannot establish harm; the GENEVA trials support the efficacy of dexamethasone implant in retinal vein occlusion, and this extract lacks adverse-event surveillance [5].

Longer-term literature underscores both the potential durability of visual benefit and the need for structured surveillance. A systematic review by Hunter and Williams synthesised outcomes from multiple retinal vein occlusion cohorts and found persisting average visual gains during long-term treatment, whilst emphasising heterogeneous study methods [13]. Rezar and colleagues described multi-year anti-VEGF management in a small BRVO series with treatment needs that changed over time [14]. In our cohort, four records had more than 60 months of follow-up, but most had substantially shorter observation; describing the entire sample as a five-year outcome cohort would therefore exaggerate the evidence. Prospective service evaluation would benefit from patient-linked records; documented treatment changes and indications; fixed 6-, 12-, and 24-month visits; both central and multi-field OCT parameters; and a prespecified safety dataset.

Clinical meaning of the magnitude and variability of visual recovery

A 12.3 ± 16.7-letter mean gain is clinically encouraging but is not equivalent to a uniformly successful course. The 42 of 102 records (41.2%) meeting the 15-letter improvement threshold and the four (3.9%) with losses of at least 15 letters demonstrate marked heterogeneity that a group mean obscures. Even the final cohort mean of 72.3 ± 13.8 recorded letters cannot be translated confidently into a standard Snellen or ETDRS (Early Treatment Diabetic Retinopathy Study) endpoint without verification of the VA measurement protocol. Some patients may gain few letters because they start with relatively good acuity, whereas others may gain many letters yet remain functionally limited. The baseline-to-final change, the final acuity achieved, and the patient’s actual ability to read, drive, or perform daily tasks are distinct clinical outcomes; this extract evaluates only the first two on its recorded letter scale.

Drug-specific interpretation benefits from evidence that directly compares agents. In a 2024 systematic review and meta-analysis comprising three randomised trials and 11 real-world studies across retinal vein occlusion types, Wu and colleagues found no statistically significant difference in BRVO visual efficacy between aflibercept and ranibizumab, although real-world BRVO cohorts recorded fewer aflibercept injections at 12 months [15]. In an international Fight Retinal Blindness! registry study of 760 treatment-naïve BRVO eyes, Alforja and colleagues reported an adjusted three-year visual gain of 11 letters (95% CI, 9-13) and 16 median injections over three years; visual outcomes were similar amongst initiating VEGF inhibitors, despite greater central-subfield thickness reduction and fewer subsequent switches in eyes starting aflibercept [16]. These results are more informative for drug selection than the unadjusted 16.8 ± 15.8-letter gain in our ranibizumab category versus 10.6 ± 16.5 in our aflibercept category. Our categories also have different starting acuities, follow-up durations, and potentially treatment histories; neither the direction nor the numerical magnitude of this between-category difference is evidence of comparative efficacy.

The distinction between a successful short-term response and durable long-term vision is especially important in an NHS setting. Dinah and colleagues analysed electronic medical records from 16 English NHS ophthalmology sites, including 3,511 BRVO eyes: mean visual change was approximately +11.2 letters at month 6 but +8.3 letters amongst the observed eyes at month 60, whilst median annual anti-VEGF injections fell from seven by month 12 to two in the fifth year [17]. Those are longitudinal summaries from a much larger dataset, not a direct control group for this study. Nevertheless, they highlight the possible effects of changing disease activity, undertreatment, and selective retention of patients in follow-up. Our median seven recorded injections cannot be treated as seven injections per year, because total recorded follow-up ranged from two to 73 months and injection-log completeness was not validated. Dose density, gaps between visits, reactivations, and reasons for discharge would be needed to distinguish low treatment requirements from missed treatment opportunities.

Retinal ischaemia, photoreceptor integrity, and structure-function dissociation

The moderate correlation between visual gain and five-field average OCT thickness reduction (Pearson r = 0.54; Spearman ρ = 0.59) is biologically plausible: resolution of intraretinal fluid may improve retinal architecture and visual function. It is equally important that the correlation leaves considerable unexplained variation. Some eyes may have a relatively dry macula but reduced acuity because foveal capillary non-perfusion, disorganisation of the retinal inner layers (DRIL), ellipsoid-zone disruption, external limiting membrane damage, chronic neuronal injury, or coincident lens pathology limits recovery. Conversely, a retina that remains thicker than average does not necessarily mean that additional injections will improve useful vision. Neither perfusion status nor these microstructural markers were captured in our dataset, so such mechanisms are explanatory hypotheses rather than observations in our 102 records.

There is growing evidence that prognosis depends on more than total thickness. In a 161-eye BRVO cohort, Cunha Ferreira and colleagues found that baseline acuity and age independently predicted final VA; multiple structural OCT abnormalities were associated with worse outcomes in unadjusted analyses [18]. An externally validated 2026 study of 356 treatment-naïve eyes proposed clinically interpretable prognostic scores based first on baseline acuity and age, then on OCT features including DRIL, hyperreflective foci, and outer-retinal disruption; discrimination improved when the OCT features were added, although the external validation sample contained only 45 eyes [19]. These findings suggest that future local datasets should collect age at BRVO onset rather than merely current age, baseline best-corrected acuity, the precise interval from symptom onset to treatment, and macular structural biomarkers. We cannot retrospectively infer any of these from an undifferentiated five-field thickness average.

Perfusion imaging adds a second and distinct dimension. In 85 eyes with resolved BRVO-associated oedema, Wakabayashi and colleagues showed that better VA was associated with greater vascular perfusion and a smaller foveal avascular zone on OCT angiography, with deep capillary plexus perfusion particularly informative in their model [20]. In a separate longitudinal RVO cohort, Winegarner and colleagues found that recurrent oedema was associated with reduced vascular density and smaller visual gains; vascular density and avascular-zone measurements did not simply normalise as thickness improved [21]. These studies explain why the thickness reduction of 113.9 ± 125.2 µm in our cohort cannot be read as reversal of macular ischaemia. Fluorescein angiography or OCT angiography, reviewed alongside en-face and cross-sectional OCT and clinical examination, could identify non-perfusion or persistent foveal injury in eyes whose acuity remains unexpectedly poor after the oedema subsides.

The measurement itself also matters. Trials frequently report central subfield thickness, a measurement centred on the fovea; our source provides a five-field average whose precise segmentation boundaries and device were not documented. The mean reduction across several retinal regions may conceal a small but visually consequential pocket of foveal fluid, whilst peripheral changes within the scanned fields may influence the average without proportionate impact on acuity. Therefore, external OCT reductions quoted in micrometres should be compared qualitatively, not numerically equated with our -113.9 µm change. Reproducible future reports should state OCT instrument and software version, field definition, central subfield thickness, intraretinal or subretinal fluid status, outer retinal band integrity, and scan quality; a masked structural grading protocol would be preferable to retrospective impressionistic classification.

Individualising anti-VEGF selection and retreatment

Current therapeutic choices extend beyond the agents represented in this historical extract. In the phase III BALATON trial of treatment-naïve BRVO-associated macular oedema (553 participants), faricimab, a dual angiopoietin-2/VEGF-A inhibitor, was non-inferior to aflibercept for visual gain at 24 weeks (+16.9 versus +17.5 adjusted letters); central subfield thinning was comparable, and a greater proportion of faricimab-treated eyes had no angiographic macular leakage at week 24 [22]. Subsequent BALATON/COMINO follow-up assessed faricimab using an individualised treat-and-extend regimen after week 24, with visual improvement broadly maintained through week 72; in BALATON, 64.1% of those initially assigned faricimab and 56.9% initially assigned aflibercept reached dosing intervals of at least 12 weeks at week 68 [23]. The extension was not a continuing randomised faricimab-versus-aflibercept comparison, as both original arms received faricimab after week 24. These findings support discussing anatomical activity and likely attendance burden together, but provide no basis to extrapolate a faricimab effect to patients in our historical dataset.

Treatment intensity must be interpreted through disease activity, not merely total injections. In the randomised SHORE study, selected patients whose RVO-associated oedema had stabilised after at least seven monthly ranibizumab injections maintained similar visual outcomes with a protocolised as-needed strategy compared with continuing monthly injections; patients who never met stability criteria behaved differently [24]. Thus, a low injection total can describe either remission or undertreatment, depending on the response history and surveillance frequency. In our study, the median follow-up of 23 months alongside a median of seven recorded injections is insufficient to classify any eye as appropriately treated, refractory, or successfully discharged. A clinically useful audit would capture loading doses, planned interval, evidence of persistent or recurrent fluid, missed appointments, the number and duration of treatment pauses, and the visual consequences of restarting therapy.

When to consider steroid treatment and what laser can and cannot achieve

Steroid-containing pathways in our data may reflect poor response to anti-VEGF, a desire to reduce visit frequency, or clinical preferences; the extract does not record these indications. Dexamethasone can reduce inflammatory and permeability-related oedema, but its effects and adverse-event profile differ from an anti-VEGF regimen. In COMRADE-B, the randomised comparison of ranibizumab with a single dexamethasone implant showed visual benefit in both groups early, followed by attenuation of benefit in the dexamethasone group after month 3; during months 3-6, mean gain was +16.2 ± 11.0 letters with ranibizumab and +9.3 ± 10.1 letters with dexamethasone [25]. This was a comparison against the specified single-implant strategy, not proof that all optimised steroid regimens are inferior. Earlier SCORE trial data also demonstrated why corticosteroid selection requires attention to raised intraocular pressure and cataract, particularly with higher-dose intravitreal triamcinolone [26]. The two dexamethasone-only records in our dataset (mean change -6.5 ± 4.9 letters) are far too few to estimate effectiveness or safety. Lens status, glaucoma risk, pressure measurements, and whether retreatment was offered would all be important before interpreting that apparent decline.

Laser has at least two distinct purposes in BRVO that should not be conflated: limited use for selected persistent macular oedema and sectoral photocoagulation for retinal neovascularisation arising from peripheral non-perfusion. The randomised RELATE trial tested adjunctive scatter/grid photocoagulation after ranibizumab and did not show a reduction in macular oedema or injection burden [27], consistent with BRIGHTER and the observational comparisons already discussed [4,12]. Yet, this does not eliminate the role of sector laser for documented new vessels; the Royal College guideline distinguishes neovascularisation surveillance and sector photocoagulation from management of centre-involving macular oedema [1]. Because the audit’s three laser-only and other small laser-containing categories do not specify the indication, timing, retinal ischaemia, or use of adjunctive therapies, they cannot answer whether laser helped control proliferative complications or reduced oedema.

Chronic disease, recurrence, longer-term risk, and the outpatient-service question

BRVO is often treated as an acute event, but the clinical burden can extend for years. In a five-year observational cohort of 97 BRVO eyes, Kiliç Müftüoğlu and colleagues reported a mean total of 19 ± 13.35 anti-VEGF injections and found that approximately one-third of eyes achieved a three-line visual improvement at five years; the retrospective design and differences in treatment exposure constrain generalisation [28]. An NHS tertiary-centre study of 757 BRVO patients within a wider retinal vascular disease cohort likewise found that both baseline acuity and retinal-fluid features influenced long-term treatment outcomes under a defined composite failure endpoint [29]. These studies help frame persistent and recurrent disease without implying that the same events occurred in our cohort. We have four eye records followed for longer than 60 months, but no fixed longitudinal sampling, relapse dates, or confirmed treatment-free periods. Consequently, a last-visit improvement cannot be equated with sustained remission.

For clinicians, the key unanswered question after an eye improves is whether to extend therapy, monitor for relapse, or discharge. A decision based solely on a normal-looking mean OCT thickness risks missing recurrent centre-involving oedema, foveal non-perfusion, or delayed neovascularisation in an eye with substantial peripheral ischaemia. The degree of non-perfusion and presence of retinal or disc neovascularisation influence the need for continued surveillance and possible sector photocoagulation [1]. Any proposed follow-up pathway should therefore document visual symptoms, best-corrected acuity, OCT fluid activity, pressure and lens status when steroids are used, and the fundus or angiographic evidence of ischaemic complications. Patients whose vision remains poor despite anatomical improvement may warrant careful evaluation of macular perfusion and outer retinal structure rather than an automatic assumption that simply increasing injection frequency will restore vision.

Whole-patient relevance and implications for future research

BRVO is also a marker of vascular health, not only a retinal treatment episode. Royal College guidance recommends identifying and addressing atherosclerotic risk factors, especially blood pressure abnormalities, dysglycaemia, and lipid disorders, usually in conjunction with primary care; routine thrombophilia testing, anticoagulation, or antiplatelet therapy solely because of an isolated RVO is not supported by the guideline [1]. Our extract does not document blood pressure, vascular risk factors, smoking, medication use, or cardiovascular follow-up, and the project’s reported exclusion of diabetes may limit generalisability relative to unselected ophthalmology populations. A better integrated service audit would record systemic risk assessment and referral completion separately from visual outcomes, whilst recognising that an association between RVO and vascular risk does not establish that its ophthalmic treatment reduces future stroke or myocardial infarction.

The next scientifically productive phase would be a linked, time-stamped, longitudinal clinical cohort rather than an unadjusted league table of drugs. Prespecifying the unit of analysis (unique eye and unique patient), ascertaining all eligible cases with a documented denominator, recording symptom onset and baseline eligibility, and applying consistent follow-up windows at six, 12, and 24 months would improve interpretability. Treatment should be modelled as a time-varying exposure; recorded switches are often consequences of prior response and cannot simply be treated as baseline randomisation. Stratified analyses of baseline acuity, documented foveal ischaemia, initial OCT morphology, lens status, steroid-related pressure changes, true injections per patient-year, and missed visits would help determine which factors predict visual recovery and persistent need for treatment. If bilateral records exist, analyses should account for correlation within the same person. These additions would allow a more clinically meaningful account of visual prognosis, avoid inappropriate causal comparisons, and generate testable hypotheses for prospective audit or multicentre observational research.

Strengths and limitations

Strengths include recalculation of paired outcomes from all 102 available rows, explicit representation of SD and uncertainty, use of both Pearson and Spearman association measures, and transparent sensitivity analyses. However, the dataset is a single-service, retrospective extract without a concurrent comparator or source screening log. The number of eligible patients, excluded patients, and unique individuals is unknown. Natural improvement, regression to the mean, case selection, and informative follow-up may all contribute to the recorded changes. The source audit listed exclusion of several ocular comorbidities, but implementation of those rules could not be independently checked and may limit generalisability.

Follow-up varied from two to 73 months, introducing unequal opportunities for treatment, recurrence, and vision change. Loss to follow-up could not be measured. The absence of patient identifiers precluded confirmation of one unique eye record per eye, counting distinct patients, identifying bilateral cases, and adjusting CIs for within-patient clustering. Treatment sequences, cataract or macular-ischaemia status, adverse events, and injection verification were unavailable. These limitations prevent reliable comparative drug rankings, safety estimates, and claims about treatment-free remission.

## Conclusions

Across 102 BRVO eye records, VA increased by 12.3 ± 16.7 letters and five-field average OCT thickness decreased by 113.9 ± 125.2 µm across variable last-visit follow-up. Functional and anatomical changes were moderately associated. The results describe routine-care observations rather than establish a causal benefit or treatment superiority. Reproducible patient linkage, clinical eligibility verification, institutional governance documentation, and standardised longitudinal measurements will be essential to strengthen future evaluations.

## Appendices

BRVO STROBE reporting checklist

**Table 3:** Study rationale and methods

STROBE item	Page	Status and author action
1. Design and abstract	1	Reported. Retrospective design is identified in the title, and the abstract summarises methods, results, and limitations.
2. Background	2	Reported. Introduction gives the clinical rationale and relevant BRVO evidence.
3. Objectives	2	Reported. Introduction states the principal and secondary objectives. Analyses are exploratory; no prespecified hypothesis is asserted.
4. Study design	2	Reported. Methods identifies the retrospective observational design.
5. Setting and dates	2	Reported. Hospital location, baseline dates, and follow-up dates confirmed.
6. Participant selection	2-3	Reported. Confirmed diagnosis, eligibility, and consecutive sampling. Matching was not used.
7. Variables	3	Partial. Outcomes and exposure categories are described. Treatment sequence, relevant clinical predictors, and variable definitions not confirmed.
8. Measurements	3	Reported. Confirmed VA method, correction, and low-vision conversions; OCT device and field definition; comparable acquisition across groups.

**Table 4:** Analysis and results

STROBE item	Page	Status and author action
9. Bias	3-4, 8-9	Partial. Selection and confounding limitations are discussed. Add the actual collection, verification, and cohort-assembly safeguards used by the team.
10. Study size	3	Reported. All 102 supplied records were analysed; no a priori sample-size calculation is claimed.
11. Quantitative variables	3-4	Reported. Means, medians, IQRs, change scores, and the 15-letter thresholds are described. Confirm the validity of the recorded letter scale.
12. Statistical methods	3-4	Partial. Methods cover CIs, correlations, missing data, and sensitivity analyses. No treatment-effect models or interactions were fitted. Confirm independence and assess loss to follow-up.
13. Participant flow	2-4	Partial. 102 analysed records are reported. Add numbers screened, eligible, excluded, followed, and analysed, with reasons. Add a flow diagram if helpful.
14. Descriptive data	4, 6	Partial. Table *1* describes the sample and paired completeness. Follow-up distribution is given. Confirm unique patients and missingness in the source cohort.
15. Outcome observations	4-6	Reported. Baseline and final VA/OCT values, changes, and 15-letter gain/loss counts are reported at variable last visits.
16. Main estimates	4-6	Reported. Overall changes and proportions include 95% CIs. Treatment summaries are unadjusted descriptions; relative-risk estimates were not produced.

**Table 5:** Interpretations and disclosures

STROBE item	Page	Status and author action
17. Other analyses	6	Reported. Post hoc exclusions of zero-letter baseline values and follow-up shorter than 12 months are reported.
18. Key findings	7	Reported. Discussion links the principal results to the study objectives.
19. Limitations	8-9	Reported. Discusses selection, confounding, variable endpoints, clustering uncertainty, and measurement limitations. Direction or size of most biases cannot be quantified.
20. Interpretation	7-9	Reported. Interpretation considers previous evidence and limitations and avoids causal treatment ranking.
21. Generalisability	9	Reported. Single-service sampling and exclusions limit applicability to broader BRVO populations.
22. Funding	9	Partial. Obtain all funding and support details and the funder’s role, or a confirmed no-funding statement.

Based on the STROBE cohort reporting framework. This is a paraphrased reporting map, not the official checklist form.

Source: [8]. Official checklist: https://www.strobe-statement.org/checklists/
